# Background luminance effects on pupil size associated with emotion and saccade preparation

**DOI:** 10.1038/s41598-020-72954-z

**Published:** 2020-09-24

**Authors:** Yih-Giun Cherng, Talia Baird, Jui-Tai Chen, Chin-An Wang

**Affiliations:** 1 Department of Anesthesiology, Shuang Ho Hospital, Taipei Medical University, Taipei, Taiwan; 2 Department of Anesthesiology, School of Medicine, College of Medicine, Taipei Medical University, Taipei, Taiwan; 3 Schulich School and Medicine and Dentistry, Western University, London, ON Canada; 4 Research Center of Brain and Consciousness, Shuang Ho Hospital, Taipei Medical University, Taipei, Taiwan; 5 Graduate Institute of Mind, Brain, and Consciousness, Taipei Medical University, Taipei, Taiwan

**Keywords:** Neuroscience, Psychology

## Abstract

Pupil dilation is consistently evoked by affective and cognitive processing, and this dilation can result from sympathetic activation or parasympathetic inhibition. The relative contributions of the sympathetic and parasympathetic systems on the pupillary response induced by emotion and cognition may be different. Sympathetic and parasympathetic activity is regulated by global luminance level. Higher luminance levels lead to greater activation of the parasympathetic system while lower luminance levels lead to greater activation of the sympathetic system. To understand the contributions of the sympathetic and parasympathetic nervous systems to pupillary responses associated with emotion and saccade preparation, emotional auditory stimuli were presented following the fixation cue whose color indicated instruction to perform a pro- or anti-saccade while varying the background luminance level. Pupil dilation was evoked by emotional auditory stimuli and modulated by arousal level. More importantly, greater pupil dilation was observed with a dark background, compared to a bright background. In contrast, pupil dilation responses associated with saccade preparation were larger with the bright background than the dark background. Together, these results suggest that arousal-induced pupil dilation was mainly mediated by sympathetic activation, but pupil dilation related to saccade preparation was primarily mediated by parasympathetic inhibition.

## Introduction

Over the past decade, there has been a resurgence of interest in pupillometry as, in addition to the illumination-dependent modulation, pupil size is an effective index for affective and cognitive processing. Pupil size is controlled by the balanced activity of the sympathetic and parasympathetic nervous systems, respectively innervating the dilator and sphincter pupillae muscles^1^. Sympathetic and parasympathetic activation lead to pupil dilation and constriction respectively^2^. Pupil dilation is observed during affective and cognitive processing^3,4^, this response may be mainly driven by sympathetic activation or parasympathetic inhibition (or reduced activation). Changing ambient light intensity allows us to understand the relative contributions of the sympathetic and parasympathetic pathways on pupillary responses as it regulates sympathetic and parasympathetic activity^5^. At lower luminance levels, there is lower activation in the parasympathetic system^5,6^. Therefore, pupil dilation driven by parasympathetic inhibition would be greatly reduced in darkness.

Pupil size has long been linked to arousal^7^, and emotional stimuli are often used to evoke arousal as the pupil dilates in response to affective processing^4,8,9^. Research using emotional pictures with concurrent recordings of pupil size, skin conductance and heart rate has demonstrated larger pupil dilation responses when viewing emotional arousing pictures^10^. Moreover, these pupil size changes covary with changes in skin conductance, a peripheral index for the sympathetic activity^11^, further suggesting that the modulation of pupil size during affecting processing is mediated by the sympathetic nervous system. Although this study importantly suggests the involvement of the sympathetic system on pupillary responses related to affective processing, factors that would influence pupil size including low-level visual properties^12–15^ and eye movements^16–19^ are not adequately controlled, and may have influenced the effects observed on pupil size during emotional picture viewing.

Pupil dilation is also reported during cognitive processing^20,21^, with larger pupil dilation observed under demanding task conditions^3,22^. A study involving varying global luminance levels during the performance of a numerical task with two difficulty levels (add 1 vs subtract 7) has demonstrated larger pupil dilation in the light condition than in the dark condition^23^. Moreover, similar results are obtained when the parasympathetic sphincter muscle is intact while the sympathetic dilator muscle is blocked, together suggesting that pupil dilation related to task difficulty is mainly mediated by inhibition of the parasympathetic pathway. These results also highlight that varying the global luminance level is an effective approach to examine the contributions of the sympathetic and parasympathetic pathways to pupillary responses. Additionally, pupil dilation responses are also demonstrated during active saccade preparation^24–26^. Research has shown larger pupil dilation responses in preparation for anti-saccades, compared to pro-saccades, prior to the saccade execution^25,26^. However, the contribution of the sympathetic and parasympathetic pathways on the observed pupil dilation responses related to saccade preparation remains unknown.

The goal of this study is to understand the relative contributions of the sympathetic and parasympathetic nervous systems to pupillary responses induced by emotional arousal and saccade preparation by varying background luminance level. We hypothesize that the effect of background luminance level on pupil size differs in the contexts of affective processing and saccade preparation, as pupil dilation associated with affective processing is mainly mediated by sympathetic excitation, while parasympathetic inhibition mainly mediates that linked to saccade preparation. Our results show larger pupil dilation associated with emotional sounds in the dark condition, but larger pupil dilation responses associated with saccade preparation in the bright condition, together revealing differences in sympathetic and parasympathetic contributions to arousal- and cognition-induced pupillary responses.

## Results

### Background luminance modulation of pupil size

**Figure 1:**
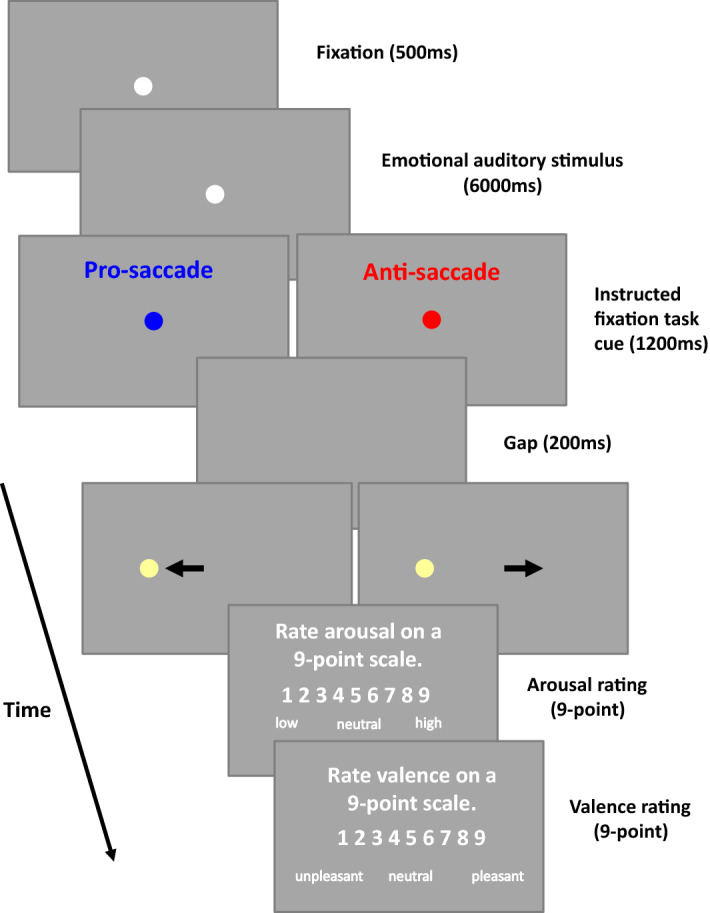
Experimental paradigm. Each trial began with a central fixation point on a background (Dark: 2 cd/m^2^ or Bright: 16 cd/m^2^). After a delay, an emotional auditory stimulus was presented for 6 s, following with the instructed colored fixation cue (1200 ms) for pro- or anti-saccade condition. A blank screen was presented for 200 ms (gap) before target stimulus presentation, and participants were required to move their eyes to the target in the pro-saccade condition, or look away to the opposite location in the anti-saccade condition, after disappearance of the central fixation point. After that, participants were required to answer two questions about the sound presented.

**Figure 2:**
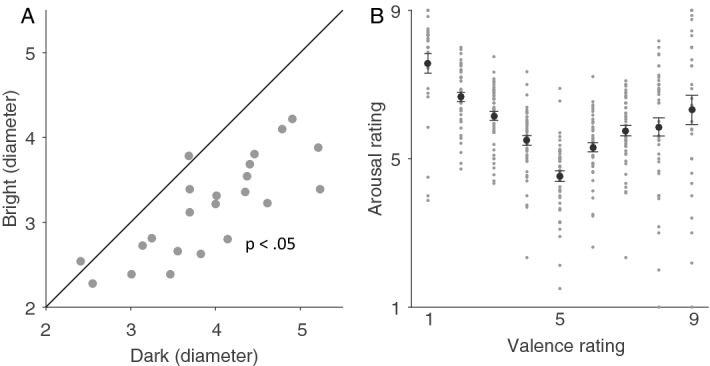
Background luminance effects on raw pupil diameter (**A**) (n = 23). Relationships between arousal and valence rating (**B**). In B, the large-circle and error-bars represent the mean values ± standard error across participants and the small circles represent mean value for each participant.

To understand the contributions of parasympathetic and sympathetic nervous systems to pupillary responses evoked by affective processing and saccade preparation, emotional auditory stimuli (International Affective Digital Sounds, IADS)^27^ were presented following a colored fixation cue indicating a pro- or anti-saccade task under two levels of background luminance (Fig. 1, see Methods for details). Consistent with the literature^15,28,29^, varying background luminance level significantly changed pupil size. Pupil size was significantly larger in the dark, compared to the bright, background (Fig. 2A, raw pupil diameter at 2000–4000 ms of post-auditory stimulus onset, mean ± s.e.m: dark: 3.946 ± 0.161 mm; bright: 3.185 ± 0.120 mm; t(22) = 7.771, p < 0.001, d = 1.620). Moreover, similar to the results using emotional faces e.g.,^30^, there was a V-shaped relationship of emotional arousal and valence rating, with higher arousal levels observed with more negative or positive sounds (Fig. 2B, arousal rating: 1 to 9 valence: 7.57 ± 0.26, 6.66 ± 0.13, 6.15 ± 0.12, 5.49 ± 0.13, 4.53 ± 0.14, 5.31 ± 0.12, 5.76 ± 0.14, 5.86 ± 0.24, 6.32 ± 0.39).

### Effects of background luminance on arousal’s modulation of pupil size

**Figure 3:**
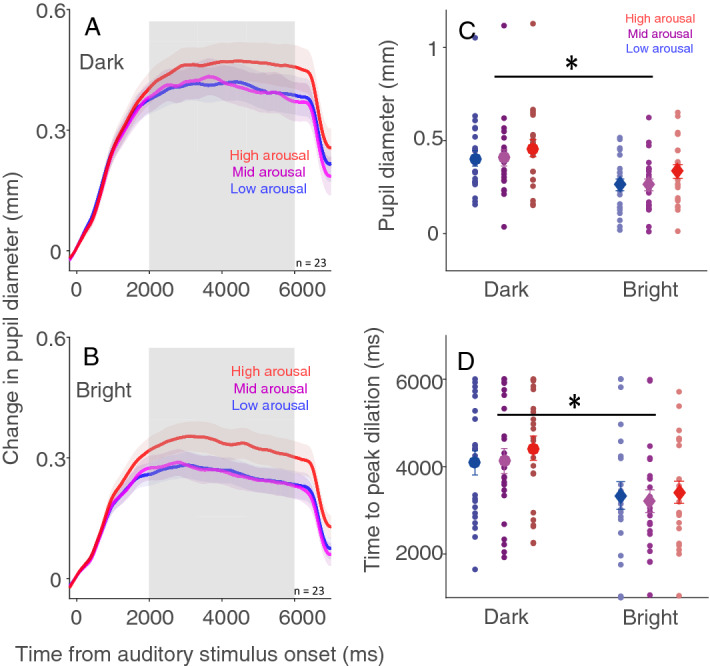
Effect of background luminance on pupillary responses associated with emotional arousal. Pupil dynamics following emotional sounds in different arousal conditions on dark (**A**) and, bright (**B**) backgrounds (n = 23). Mean pupil size (2000 to 6000 ms) (**C**) and time to peak dilation (**D**) of pupillary responses can be seen in different arousal conditions with dark and bright backgrounds. In A and B the shaded colored regions surrounding the pupillary response curves represent the ± standard error range (across participants) for different conditions. In **C** and **D**, the large-circle and error-bars represent the mean values ± standard error across participants. The small circles represent mean value for each participant. *Indicates differences are statistically significant.

If pupil dilation evoked by emotional arousal is mainly mediated by sympathetic activation, increasing background luminance level would increase parasympathetic activation and in turn reciprocally inhibit sympathetic activation. Emotional auditory stimuli would then evoke more limited pupil dilation responses in the bright condition, as compared to the dark condition. If, however, arousal-induced pupil dilation is mainly mediated by parasympathetic inhibition, decreasing background luminance level would decrease parasympathetic activation, and therefore reduce parasympathetic inhibition, resulting in less pupil dilation in the dark condition. To investigate the influence of background luminance level on the pupil response induced by emotional arousal, we separated trials into three arousal categories (high: arousal > the median arousal value of a given subject; mid: arousal = the median arousal value of a given subject; low: arousal < the median arousal value of a given subject) according to the subjective arousal ratings. Consistent with previous studies^4,8,9^, pupil dilation was evoked by emotional auditory stimuli (Fig. 3A and B). Mean pupil sizes (2000 – 6000 ms of post-stimulus onset) with the dark background were 0.404 ± 0.041, 0.405 ± 0.043, and 0.459 ± 0.045 mm in the low, mid, and high arousal conditions respectively, and were 0.262 ± 0.031, 0.261 ± 0.031, and 0.334 ± 0.036 mm with the bright background in the low, mid, and high arousal conditions respectively (similar patterns were observed in peak dilation in supplementary Fig. 1). Significantly greater pupil dilation was observed with the dark, compared to the bright background (Fig. 3C, F(1,22) = 25.051, *p* < 0.001, η^2^ = 0.125), and stimuli with higher arousal levels evoked greater pupil dilation (F(2,44) = 14.598, *p* < 0.001, η^2^ = 0.024). The interaction of background luminance and arousal level was not significant (F(2,44) = 0.658, *p* = 0.515, η^2^ = 0.00). Furthermore, time to peak dilation was longer in the dark, compared to, the bright condition (Fig. 3D, F(1,22) = 9.079, *p* = 0.006, η^2^ = 0.107), with mean times of 4084 ± 270, 4126 ± 283, and 4422 ± 273 ms with the dark background in the low, mid, and high arousal conditions, respectively, and 3339 ± 309, 3208 ± 254, and 3412 ± 248 ms with the bright background in the low, mid, and high arousal conditions, respectively. Main effects of arousal and the interaction of background luminance and arousal level were not significant (arousal: F(2,44) = 1.355, *p* = 0.269, η^2^ = 0.006; interaction: F(2,44) = 0.336, *p* = 0.716, η^2^ = 0.002). Notably, a regression model was used to investigate this modulation, using arousal level as continuous variable. These results revealed the same effects (see details in supplementary information Fig. 1). Together, these results suggested that pupil dilation related to emotional arousal was mainly mediated by the sympathetic pathway. Pupil valence modulation findings are included in the supplementary materials because, as demonstrated in the literature, the modulation of valence on pupil size was less reliable^9,10^ (supplementary Fig. 2).

### Background luminance level modulation of pupil responses during pro- and anti-saccade preparation

**Figure 4:**
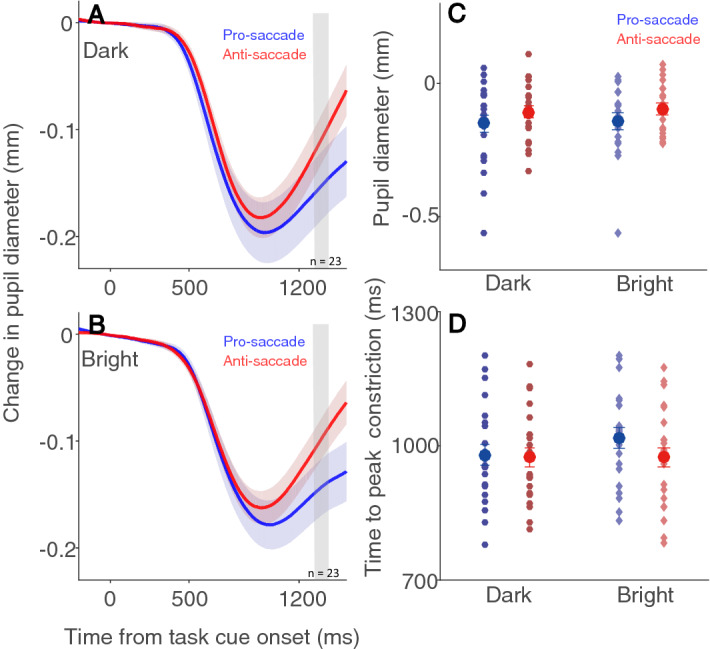
Effect of background luminance on pupillary responses related to saccade preparation. Pupil dynamics following instructed fixation cue in different saccade preparation conditions on dark (**A**) and, bright (**B**) backgrounds (n = 23). Mean pupil size (2000 to 6000 ms) (**C**) and time to peak dilation (**D**) of pupillary responses can be seen in different saccade preparation conditions with dark and bright backgrounds. In (**A**) and (**B**) the shaded colored regions surrounding the pupillary response curves represent the ± standard error range (across participants) for different conditions. In C and D the large-circle and error-bars represent the mean values ± standard error across participants. The small circles represent mean value for each participant.

**Figure 5:**
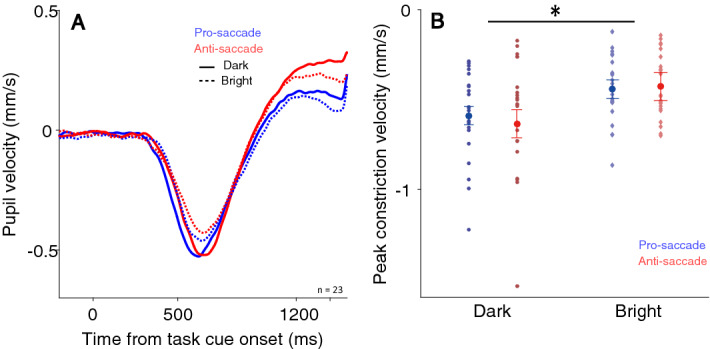
Effect of background luminance on pupil velocity responses related to saccade preparation. (**A**) Pupil velocity dynamics following the instructed fixation cue in different saccade preparation conditions between the dark and bright background. (**B**) Peak pupil constriction velocity in different saccade preparation conditions with dark and bright backgrounds. In (**A**), the shaded colored regions surrounding the pupillary response curves represent the ± standard error range (across participants) for different conditions. In (**B**), the large-circle and error-bars represent the mean values ± standard error across participants. The small circles represent mean value for each participant. *Indicates differences are statistically significant.

To investigate the influence of background luminance level on pupil size associated with saccade preparation^24–26^, pupil size was baseline-corrected to the onset of the instructed fixation cue (see Methods). If the pupillary response linked to saccade preparation is mainly mediated by parasympathetic inhibition, pupil dilation responses (smaller early peak constriction and larger late pupil dilation) should be larger with the bright than with the dark background due to greater parasympathetic inhibition with the bright background. The pupil initially constricted after the instructed fixation period regardless of background luminance (Fig. 4A,B). As the luminance level was unchanged during the whole trial period, this initial constriction likely resulted from transient changes in foveal vision, as previously demonstrated^12^. To quantify pupillary responses, mean pupil size around target onset (100 ms before to target onset) was analysed, showing significantly larger pupil size in the anti-saccade condition than in the pro-saccade condition (Fig. 4C, F(1,22) = 11.097, *p* = 0.003, η^2^ = 0.037), with mean pupil sizes of -0.15 ± 0.031 and -0.106 ± 0.022 mm with the dark background in the pro- and anti-saccade conditions, and -0.143 ± 0.026 and -0.096 ± 0.019 mm with the bright background in the pro- and anti-saccade conditions. Though pupil size was larger in the bright than in the dark condition (similar patterns were observed in peak constriction in supplementary Fig. 3), the differences were not statistically significant (F(1,22) = 0.378, *p* = 0.545, η^2^ = 0.001). Other effects were negligible (*p* > 0.77). Times to peak constriction were 980 ± 23 and 974 ± 21 ms with the dark background, and 1018 ± 21 and 974 ± 22 ms with the bright background in the pro- and anti-saccade conditions respectively (Fig. 4D). All other effects failed to reach significance (all *ps* > 0.08). Instantaneous changes in pupil size (pupil velocity, Fig. 5A) revealed significantly smaller peak constriction velocities with the bright than with the dark background (Fig. 5B, F(1,22) = 11.014, *p* = 0.003, η^2^ = 0.113), with mean velocities of 0.587 ± 0.005 and -0.634 ± 0.077 mm/s with the dark background, and -0.440 ± 0.037 and -0.427 ± 0.037 mm/s with the bright background in the pro- and anti-saccade conditions respectively. Other effects were negligible (all *ps* > 0.16). These results suggested that stronger parasympathetic inhibition, producing a greater reduction in peak constriction velocities in the bright than in the dark condition, was the primary mediator of pupil dilation associated with saccade preparation.

### Comparing the effects of background luminance on pupillary responses associated with emotional arousal and saccade preparation

**Figure 6:**
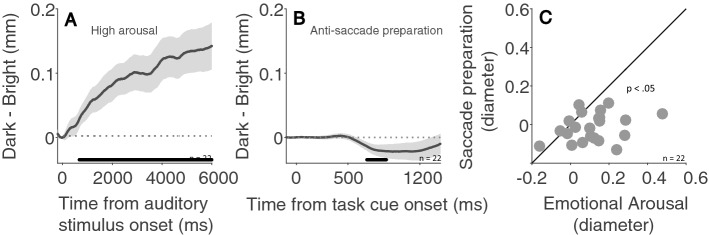
Effect of background luminance on pupillary responses induced by emotion and saccade preparation. (**A**) Difference between pupillary responses evoked by negative valence stimuli with dark and bright backgrounds (dark minus bright pupil size) (n = 21). (**B**) Difference between pupillary responses related to anti-saccade preparation with dark and bright backgrounds (dark minus bright pupil size) (n = 21). (**C**) Mean participant differences in pupil size between dark and bright backgrounds (dark minus bright pupil size) related to emotion and saccade preparation (emotion: 2000—6000 ms; saccade preparation: 1300–1400 ms). In (**A**,**B**), the shaded regions surrounding the pupillary response curves represent the ± standard error range (across participants). The black bar on the x-axis indicates the time line at which differences between the dark and bright conditions were statistically deviated from zero (P < 0.05).

To compare the influence of background luminance level on pupillary responses associated with arousal and saccade preparation, we compared pupillary responses in the dark and bright conditions associated with affective processing (high arousal level) and saccade preparation (anti-saccade). Figure 6A illustrates differences in pupillary responses (dark minus bright pupil size) following highly arousing sounds, the increase in pupil size was larger in the dark than in the bright condition. In contrast, pupil size associated with saccade preparation was generally smaller in the dark than in the bright condition, as illustrated in Fig. 6B (dark minus bright pupil size in the anti-saccade condition), though the effects were not always statistically significant. Differences in pupil size between dark and bright conditions were significantly larger with emotional arousal than with saccade preparation (Fig. 6C, mean difference pupil size in the epoch of 2000—6000 ms after emotional stimuli: 0.137 ± 0.029; mean difference pupil size in the epoch of 1300—1400 ms after the instructed fixation cue: -0.013 ± 0.015; t(21) = 4.635, p < 0.001, *d* = 0.988). This suggested that autonomic modulation of pupil dilation in response to emotional sounds and saccade preparation differs.

### Background luminance effects on saccade behavior

To examine the influence of background luminance level on saccade behavior, we analyzed direction error and saccade reaction times (SRT) with two background luminance levels. Mean error rates were 0.409 ± 0.201 and 16.297 ± 2.143% with the dark background and 0.714 ± 0.235 and 17.910 ± 2.121% with the bright background, in the pro- and anti-saccade conditions respectively (Supplementary Fig. 4A). Consistent with the literature^31–35^, saccade preparation significantly affected task accuracy, with more direction errors in the anti-saccade than in the pro-saccade condition (F(1,22) = 74.114, *p* < 0.001, η^2^ = 0.574). Background luminance level did not modulate direction error rates (F(1,22) = 0.913, *p* = 0.350, η^2^ = 0.002). Other effects were negligible (*p* > 0.53). Mean SRTs were 146 ± 6.001 and 203 ± 4.742 ms with the dark background, and 144 ± 6.535 and 201 ± 5.629 ms with the bright background, in the pro- and anti-saccade conditions respectively (Supplementary Fig. 4B). Similarly, longer SRT were observed in the anti-saccade than the pro-saccade condition (F(1,22) = 180.943, *p* < 0.001, η^2^ = 0.529). Other effects were negligible (all *ps* > 0.57). Given that saccade behavior was similar in the dark and bright conditions, these data were collapsed for subsequent analyses.

### Arousal modulation of saccade behavior

**Figure 7:**
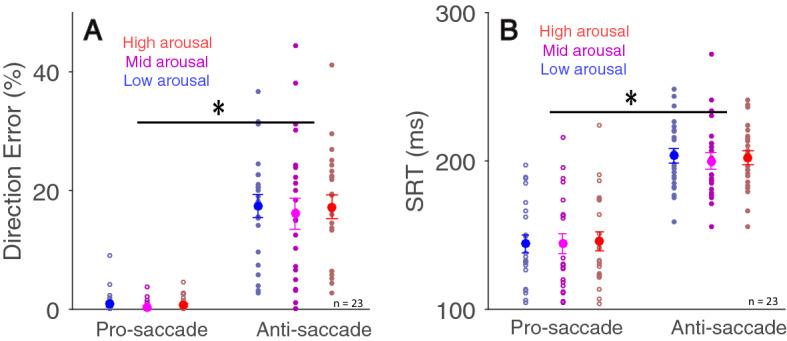
Effects of emotional arousal on saccade behavior. Saccade direction error (**A**) and saccade reaction times (**B**) during pro- and anti-saccade tasks with different arousal conditions (n = 23). The large-circle and error-bars represent the mean values ± standard error across participants. The small circles represent mean value for each participant. *Indicates differences are statistically significant.

To examine the influence of emotional arousal on saccade behavior^36,37^, we separated trials into three arousal or valence categories and collapsed two background luminance conditions (same as Fig. 2 analyses). Mean direction error rates in pro-saccade trials were 0.828 ± 0.046, 0.372 ± 0.196, and 0.693 ± 0.271% in the low, mid, and high arousal conditions respectively, and were 17.392 ± 1.907, 16.086 ± 2.570, and 17.270 ± 1.970% in anti-saccade trials in the low, mid, and high arousal conditions respectively (Fig. 7A). Although saccade preparation significantly modulated accuracy (F(1,22) = 72.418, *p* < 0.001, η^2^ = 0.555), emotional arousal did not affect directional error rates (F(1,22) = 0.453, *p* = 0.639, η^2^ = 0.001). The interaction of task and emotional arousal was not significant (F(2,44) = 0.131, *p* = 0.877, η^2^ = 0.000). Similarly, SRT was only modulated by saccade preparation (Fig. 7B, F(1,22) = 156.789, *p* < 0.001, η^2^ = 0.537), with mean SRTs in pro-saccade trials of 144 ± 5.861, 144 ± 6.544, and 146 ± 6.277 ms, and 203 ± 4.866, 200 ± 5.504, and 202 ± 4.617 ms in anti-saccade trials in the low, mid, and high arousal conditions respectively. No effects reached significance (all *ps* > 0.47). Valence effect findings are appended in supplementary Fig. 5.

## Discussion

The current study examined the effects of background luminance level on pupillary responses associated with affective processing and saccade preparation. Pupil dilation was evoked by emotional sounds and modulated by emotional arousal under different levels of background luminance. Importantly, larger pupil dilation was observed in the dark than in the bright condition, suggesting that arousal-induced pupil dilation is mainly produced through sympathetic activation. In contrast, a larger pupil dilation response was observed with higher background luminance during saccade preparation, with reduced peak constriction velocities in the bright condition, compared to the dark condition, suggesting that this response was mediated by parasympathetic inhibition. The contrast in background luminance effects demonstrated the difference in relative sympathetic and parasympathetic contributions to arousal- and saccade-preparation-induced pupillary responses.

The contributions of the sympathetic and parasympathetic branches in the autonomic nervous system to pupil size has been investigated previously^23,38–40^. Early study investigating pupil dilation evoked by auditory stimuli (pure tone presented for 200 ms) has demonstrated the effects of changes in illumination level on pupil dynamics following auditory stimuli^40^. Though the magnitude of peak pupil dilation is unchanged across illumination levels, two peaks are observed in high illumination levels, suggesting the involvement of parasympathetic inhibition in the high illumination condition. Here, consistent with the findings of other studies using emotional sounds^8,9^, pupil dilation was modulated by emotional arousal (Fig. 3). Further, larger pupil dilation was observed in the dark than in the bright condition (Fig. 6A), suggesting the major involvement of the sympathetic system in arousal-induced pupil dilation. Our results corresponded to those of other studies using emotional pictures, showing that pupil dilation responses during emotional picture viewing covary with sympathetic activity revealed by skin conductance^10^. Together, these results provide converging evidence to support the modulation of arousal-induced pupil dilation mainly by the sympathetic nervous system.

To investigate the sympathetic and parasympathetic contributions to changes in pupil size related to task difficulty, a seminal study has varied illumination level and pharmacologically blocked sphincter or dilator muscles^23^. Larger pupil dilation is observed during sustained cognitive processing in the bright condition than in the dark condition, and this effect is greatly eliminated by blocking parasympathetic sphincter muscle activity. These results suggest that pupillary dilation associated with task difficulty is primarily mediated by parasympathetic inhibition. Moreover, as the pupillary light reflex is mediated mainly by the parasympathetic pathway^1^, supressed parasympathetic activity during demanding cognitive processing should reduce the pupil light reflex responses. Studies that examine the influence of cognitive processing on the pupil light reflex have further confirmed this idea, showing reduced pupillary light reflex responses during demanding cognitive processing^41,42^. Here, we found larger pupil dilation in preparation for anti-saccades than pro-saccades (Fig. 4), consistent with previous studies^25,26^. Moreover, pupil dilation responses associated with saccade preparation were larger in the bright than in the dark condition (Fig. 5 & 6B), suggesting that these dilation responses were primarily due to parasympathetic inhibition. Notably, there is a degree of inconsistency, as some studies manipulating task difficulty have shown larger pupil dilation in the bright than in the dark luminance condition^38,39,43^. Future studies are needed to investigate the effects of various types of task difficulty on relative sympathetic and parasympathetic contributions to evoked pupillary responses.

Pupil dilation is linked to mental effort in cognitive control tasks^22^, and emotional processing can affect mental effort and in turn increase pupil size (e.g.,^44^), therefore both arousal and cognition pupil modulation may be driven by mental effort. Mental effort however is a broad concept, and mental effort cannot explain all associated pupil dilation effects, and thus it is possible that different neural mechanisms are involved in different types of pupil dilation effects related to mental effort. For example, a study of varying task difficulty has shown smaller pupil dilation (phasic) in the difficult condition than in the easy condition^39^. Our previous study in saccade preparation^25,26^ has also suggested that mental effort (or processing resources) cannot fully explain our pupil results related to saccade preparation in the interleaved pro- and anti-saccade paradigm (detailed in^26^ Discussion 4.3). Moreover, both sympathetic-activation and parasympathetic-inhibition are likely involved in different types of pupil dilation responses, due to the reciprocal relationship between the sympathetic and parasympathetic nervous systems. Although some pupil modulations are mainly mediated by either pathway, another pathway can still affect these pupil responses. For example, pupil light reflex responses, though mainly mediated by the parasympathetic system, are reduced during affective processing^45,46^, and these results are likely mediated by inhibitory connections from the sympathetic to parasympathetic system.

Could the pupillary changes described may be a response to luminance level due to the short presentation of the auditory stimuli following central fixation? The pupil may still be changing in response to background luminance after central fixation, resulting in larger pupil dilation in the dark than in the bright background. As the background luminance level was reset during the inter-trial-interval period before central fixation point presentation, the pupil has already reached to a rather stable state before FP appearance. Further, differences in pupil size between the dark and bright conditions were already pronounced at fixation onset (supplementary Fig. 6). It is thus unlikely that the pupil darkness reflex was pronouncedly involved after central fixation, affecting arousal-induced pupil dilation. Additionally, because raw pupil diameter was larger with dark background than with bright background, it can be argued that there is more room for the pupil to dilate in the bright than in the dark background, which could result in larger pupil dilation responses associated with saccade preparation in the bright than in the dark background condition. Although this is possible, we believe that this is unlikely as pupil diameter can increase up to 7–8 mm, and the upper bound of pupil diameter here ranged from 3 to 5 mm. Therefore, there is still plenty of room for the pupil to dilate even in the dark background condition.

Which neural substrates mediate this behavior? The reciprocal relationship between the sympathetic and parasympathetic nervous systems is generally accepted, and that the sympathetic and parasympathetic pupil control systems have mutually inhibitory cross connections. There is inhibitory input from the hypothalamus to the Edinger–Westphal (EW) nucleus in the parasympathetic pathway to suppress the activity of the sphincter pupillae muscle^47,48^. It is also possible that the parasympathetic system also sends inhibitory input to the sympathetic pathway to suppress the dilator pupillae muscle through direct projection from the EW to the cervical spinal cord^49,50^, though this possibility has yet to be systematically investigated.

The locus coeruleus (LC), a small brainstem nucleus involved in the regulation of arousal^51–57^, is regularly implicated in the close relationship of pupil size and emotional arousal^51,58^. Therefore, the LC is likely involved in arousal-induced pupil dilation. Although the anatomical connections between the LC and pupil control brainstem nuclei are less established, it is argued^59^ that LC-mediated pupillary responses could be produced through its connections to the hypothalamus^60^ or the common input from the nucleus paragigantocellularis of the ventral medulla to both the LC and the EW nucleus^61–63^. Additionally, the LC also projects to the intermediolateral (IML) spinal cord a part of the sympathetic nervous system (for review see^64^). As arousal-induced pupil dilation is mainly driven by sympathetic activation, it is more likely that this dilation was mediated by the pathway from the LC to the IML spinal cord and/or hypothalamus. Moreover, because sympathetic activity should be reduced in the bright condition through parasympathetic inhibition, we found that arousal-induced pupil dilation was smaller in the bright than in the dark condition.

The superior colliculus (SC), a midbrain structure causally involved in gaze and attention shifts^65–67^, is believed to coordinate pupil dilation responses associated with saccade preparation^19,25,26^. The connections between neurons in the SC and the brainstem reticular formation, to control saccades, is well-established (for review see^68^. In particular, saccade-related long-lead burst neurons with axons in the central mesencephalic reticular formation (cMRF) are usually SC efferents^69,70^, and cMRF neurons project directly to the EW nucleus^71,72^. The connections between the SC and EW nucleus via cMRF thus likely mediates pupil dilation associated with saccade preparation via parasympathetic inhibition. In summary, we argue that arousal-induced pupil dilation is mediated by the sympathetic activation through the LC-IML/hypothalamus pathway, and pupil dilation related to saccade preparation is mediated by parasympathetic inhibition through the SC-cMRF-EW pathway.

## Materials and methods

### Experimental setup

All experimental procedures were reviewed and approved by the Institutional Review Board of the Taipei Medical University, Taiwan, and were in accordance with the Declaration of Helsinki^73^. Twenty-six participants (mean age: 25.7, SD: 4 years, 13 males) were recruited via an advertisement posted on the Taipei Medical University website. Participants had normal or corrected-to-normal vision and were naïve regarding the purpose of the experiment. Participants provided informed consent and were compensated financially for their participation.

### Recording and apparatus

Participants were seated in a dark room. Eye position and pupil size were measured with a video-based eye tracker (Eyelink-1000 plus binocular-arm, SR Research, ON, Canada) at a rate of 500 Hz with binocular recording (left pupil was used), and stimulus presentation and data acquisition were controlled by Eyelink Experiment Builder and Eyelink software, as described previously^35^. Auditory stimuli were presented using headphones. Visual stimuli were presented on an LCD monitor at a screen resolution of 1920 × 1080 pixels (60 Hz refresh rate), subtending a viewing angle of 58° × 32°, with the distance from the eyes to the monitor set at 60 cm.

### Emotional sound with pro- and anti-saccade task (Fig. 1)

Each trial began with the appearance of a central fixation point (FP) (0.5° diameter, grey color with 20 cd/m^2^) on a background (Dark: 2 or Bright: 16 cd/m^2^) in a dark room with the only source of illumination from the monitor. After 500 ms of central fixation, an emotional auditory stimulus from the International Affective Digital Sounds^27^ was presented with a central FP for 6000 ms. Afterward, though the luminance level did not change, the color of the FP changed in accordance with the saccade task condition (the luminance level of the two FP colors were matched, 20 cd/m^2^; because our previous study showed similar effects between two FP color groups^35^, we did not counterbalance FP colors across participants in this study). In pro-saccade trials, participants were instructed to look towards the peripheral target stimulus as soon as the target appeared. In anti-saccade trials, participants were instructed to look in the opposite direction of the target stimulus as soon as the target appeared. After another 1200 ms of central fixation, the FP disappeared for 200 ms (gap) before the peripheral target stimulus appeared (0.5° diameter; yellowish dot with luminance 270 cd/m^2^) to the left or right of the FP (8° eccentricity on the horizontal axis). This was followed by two questions presented on the screen. The participants were asked to rate the degree of arousal and valence of the stimuli on nine-point scales and using the keypad to respond^30,74^. When rating arousal, 1 indicated a low and 9 indicated a high degree of arousal. When rating valence, 1 indicated an unpleasant stimulus whereas, 9 indicated a pleasant stimulus, with 5 representing a neutral value. The next trial commenced after an inter-trial interval of 3–4 s during which the screen’s luminance was returned to the level of stimulus presentation after a brief presentation of the black blank screen, such that the luminance level was returned to the stimulus presentation level way before the appearance of central FP. The participants came in twice (one week apart) for the same experiment with a different background luminance level (the order of two background luminance level experiments was counterbalanced across participants). To ensure that each task condition involved equal numbers of each level of emotional valence, trials were separated into three valence categories (each category had 52 trials) according to the previously-established rating values^27^. Each set of trials for a particular emotional valence contained 13 trials for each saccade and stimulus condition. The experiment consisted of 156 trials, in addition to 5 practice trials (two practice sounds not from IADS). Task condition (pro- and anti-saccade) and stimulus location (left and right) were randomly interleaved. Saccades toward either the right or left direction were combined for data analysis.

### Stimuli

Audio clips were selected from the International Affective Digitized Sound (IADS) database system^27^, and 8 clips were excluded due to short duration (< 6000 ms; clips 104, 204, 275, 296, 365, 400, 698, 699). Auditory stimuli were presented through headphones. The volume level was fixed across participants, set to a level that was predetermined to be comfortable and clear for all subjects.

### Data analysis

Saccade reaction time (SRT) was defined as the time from peripheral target appearance to the first saccade away from fixation (eye velocity exceeded 30^°^/s) with an amplitude greater than 3°. Trials were scored as correct if the first saccade after stimulus appearance was in the correct direction (toward the stimulus in the pro-saccade condition; away from the stimulus in the anti-saccade condition). Direction errors were identified as the first saccade after target appearance in the wrong direction (e.g. toward stimulus on anti-saccade trials). Trials with an eye position deviation of more than 2° from the central FP or with detected saccades (> 2° amplitude) during the required period of central fixation were excluded from analysis. Two participants were removed from analysis due to the number of blinks and presence of eye movements during the fixation period. Additionally, two participants made small eye movements (~ 3°) during emotional sound presentation in a majority of trials. As similar pupillary responses were seen in trials with and without these eye movements for these participants, these trials were not excluded. When blinks were detected, following the literature, pre- and post-blink pupil values were used to perform a linear interpolation to replace pupil values during the blink period^75–77^. Trials were discarded when two blinks occurred within a time interval of less than 500 ms. Trials in which a saccade was made prior to the disappearance of the FP or there was failure to initiate a saccade within 800 ms after the disappearance of the FP or a saccade, were excluded from the analysis. The saccades with SRTs < 90 ms were considered as anticipatory^78^ and excluded from analyses. In addition to pupil diameter data, we also computed the instantaneous pupil diameter derivative (i.e., velocity) to further examine moment-to-moment pupillary changes. Two separate baseline-correction epochs were used for emotion (100 ms prior to auditory stimulus onset to auditory stimulus onset) and saccade preparation (100 ms prior to the instructed fixation onset to the instructed fixation onset) analyses. At least 10 correct trials remained for emotional sound and saccade preparation analysis for twenty-three participants (one participant excluded from each analysis), with different participant being excluded from each analysis. Peak dilation (or constriction) was defined as the maximum (or minimum) value observed within the period of time after the stimulus onset (emotion: 2000—6000 ms; saccade preparation: 100—1200 ms). The time-to-peak response was defined as the time to the peak dilation (or constriction) response.

A two-way repeated-measure ANOVA was used to examine effects of background luminance (dark and bright) and emotional sounds (arousal or valence) on the pupillary response and Bonferroni-corrected *t*-test was used for the planned comparisons, except where indicated. We also examined effects of background luminance (dark and bright) and saccade preparation (pro- and anti-saccade) on the pupillary response. A two-tailed student *t*-test was sometimes performed to compare the differences between the two conditions. Effect sizes, where appropriate, were also reported. Statistical tests were performed using JASP Team (2019)^79^.

## Supplementary information

Supplementary file1

## Data Availability

Data are available from the Open Science Framework (https://osf.io/dhtp9/) following the publication.
