# Supplementary material for: Background luminance effects on pupil size associated with emotion and saccade preparation
Source: Sci Rep. 2020 Sep 24;10:15718. doi: 10.1038/s41598-020-72954-z (PMC7515892; doi:10.1038/s41598-020-72954-z)
Supplement: Supplementary file 1 — Supplementary file1 [file 41598_2020_72954_MOESM1_ESM.pdf]

Title:

Background luminance effects on pupil size associated with emotion and saccade preparation

Yih-Giun Cherng, Talia Baird, Jui-Tai Chen and Chin-An Wang

Supplementary materials

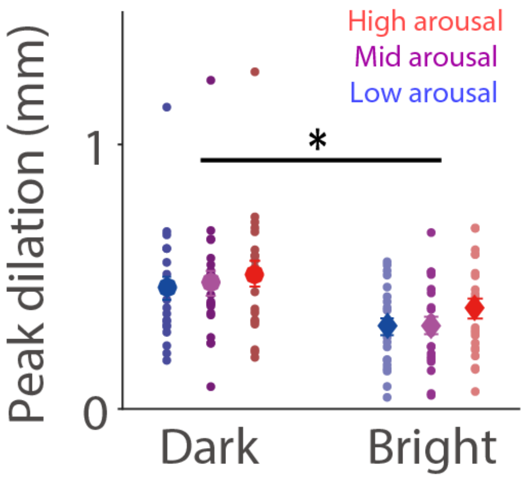

Supplementary Figure 1. Arousal modulation between two background luminance levels on peak pupil dilation. Mean pupil sizes in peak dilation were 0.456 ± 0.042, 0.475 ± 0.046, and 0.513 ± 0.048 mm in the low, mid, and high arousal conditions, respectively, and 0.309 ± 0.031, 0.317 ± 0.032, and 0.379 ± 0.037 mm with bright background in the low, mid, and high arousal conditions, respectively. Pupil dilation was larger with the dark, compared to the bright background ($F(1,22) = 24.971$, $p < 0.001$, $\eta^2 = 0.131$), and more arousing stimuli evoked larger pupil dilation ($F(2,44) = 13.107$, $p < 0.001$, $\eta^2 = 0.018$). The interaction of background luminance and arousal level was not significant ($F(2,44) = 0.721$, $p = 0.482$, $\eta^2 = 0.001$).

To further investigate the effect of arousal level on evoked pupil dilation, we performed a linear regression analysis allowing us to consider data from all trials across all subjects while using arousal rating as continuous variable. More specifically, we investigated the influence of arousal rating (1 to 9) and background luminance level (Dark: 1; Bright: 2) on mean pupil dilation based on the model using arousal (A), background luminance (L) and combined these two ($A*L$) as fixed predictors.

$$\text{Pupil dilation} = \beta_0 + \beta_1 * A + \beta_2 * L + \beta_3 * A * L \qquad (1)$$

where β_i are the standard coefficients of the statistical model (intercept and slopes).
The results yielded $\beta_1 = 0.028$, $p = 9.6e\text{-}06$; $\beta_2 = -0.11$, $p = 4.4e\text{-}06$; $\beta_3 = -0.006$, $p = 0.13$, demonstrating that pupil dilation was accounted by arousal and background luminance levels. These results were consistent with the results that split trials to different groups according to arousal rating. Together, these suggested that both arousal and background luminance level account for some fluctuations of mean pupil dilation on a trial-by-trial basis.

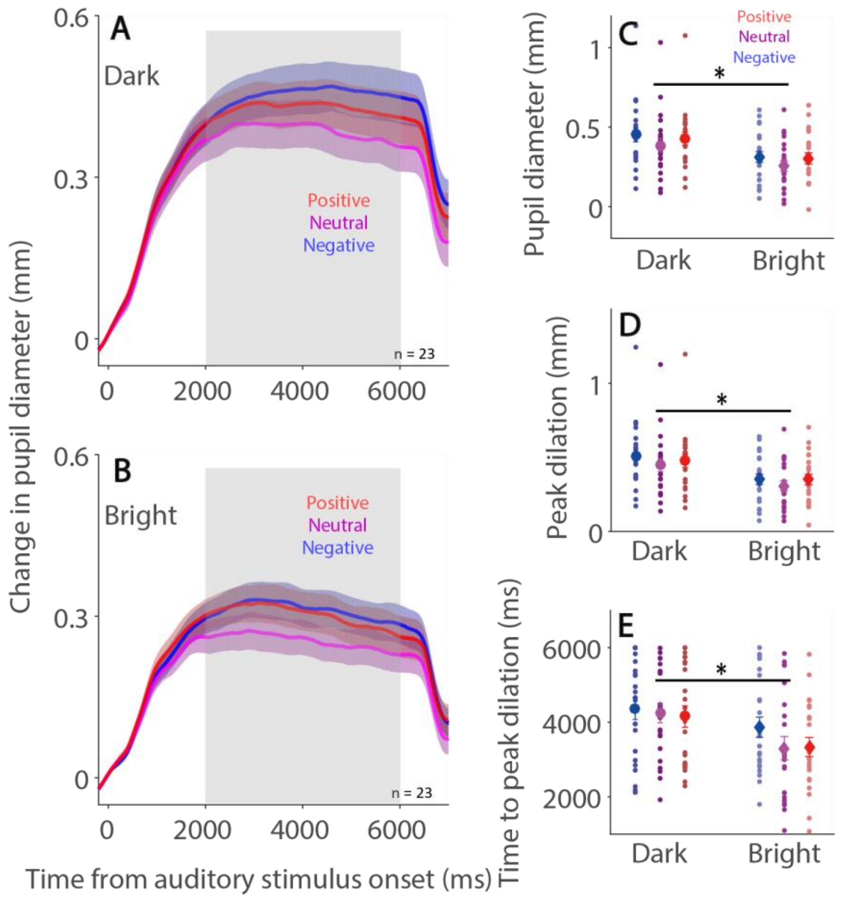

Supplementary Figure 2. Valence modulation between two background luminance levels on pupil size. To investigate the influence of background luminance level on the pupil response induced by emotional valence, we separated trials into three valence categories (positive: valence > 5; neutral: valence = 5; negative: valence < 5) according to the subjective valence ratings. The pupil dilated after the presentation of emotional auditory stimuli, regardless of background luminance level (supplementary Fig. 2A & 2B). Mean pupil sizes (2000 – 6000 ms of post-stimulus onset) with the dark background were 0.453 ± 0.044, 0.386 ± 0.042, and 0.428 ± 0.040 mm in the negative, neutral, and positive valence conditions respectively, and were 0.312 ± 0.033, 0.254 ± 0.031, and 0.304 ± 0.034 mm with the bright background in the negative, neutral, and positive valence conditions, respectively. Pupil dilation was larger with the dark than with the bright background (supplementary Fig. 2C, F(1,22) = 26.107, $p < 0.001$, $\eta^2 = 0.120$), and valence significantly modulated evoked pupillary responses (F(2,44) = 21.465, $p < 0.001$, $\eta^2 = 0.019$), with larger pupil dilation observed in negative (negative vs neutral: bright: post hoc with Bonferroni-corrected t= 4.208, p < 0.001, d = 0.878; dark: Bonferroni-corrected t= 4.904, p < 0.001, d = 1.023) or positive valence stimuli (positive vs neutral: bright: t= 3.607, p = 0.003, d =

0.752; dark: t= 3.069, p = 0.043, d = 0.64). Consistent with the literature [9,10], differences between negative and positive valence were not significant. The interaction of background luminance and valence level was not significant (F(2,44) = 0.697, $p = 0.659$, $\eta^2 = 0.000$). Similar patterns were observed in peak dilation (supplementary Fig. 2D), with mean pupil sizes of 0.504 ± 0.046, 0.447 ± 0.043, and 0.477 ± 0.043 mm in the negative, neutral, and positive valence conditions respectively, and 0.353 ± 0.033, 0.308 ± 0.033, and 0.350 ± 0.034 mm with the bright background in the negative, neutral, and positive valence conditions respectively. Pupil dilation was larger in the dark, compared to the bright background (F(1,22) = 25.371, $p < 0.001$, $\eta^2 = 0.124$), and negative (bright: t= 3.169, p = 0.032, d = 0.661; dark: t= 4.039, p = 0.002, d = 0.842) or positive (bright: t= 2.983, p = 0.056, d = 0.622; dark: t= 2.144, p = 0.52, d = 0.447) valence stimuli evoked larger pupil dilation (main valence effects: F(2,44) = 13.068, $p < 0.001$, $\eta^2 = 0.011$). The interaction of background luminance and arousal level was not significant (F(2,44) = 0.771, $p = 0.457$, $\eta^2 = 0.001$). Furthermore, time to peak dilation was longer in the dark, compared to, the bright condition (supplementary Fig. 2E, F(1,22) = 7.650, $p = 0.011$, $\eta^2 = 0.077$), with times of 4367 ± 284, 4243 ± 253, and 4155 ± 283 ms with the negative, neutral, and positive valence conditions respectively, and 3867 ± 266, 3304 ± 304, and 3337 ± 250 ms with bright background in the negative, neutral, and positive valence conditions respectively. The main effect of valence and the interaction of background luminance and arousal level were not significant (valence: F(2,44) = 2.183, $p = 0.125$, $\eta^2 = 0.016$; interaction: F(2,44) = 0.607, $p = 0.541$, $\eta^2 = 0.005$). These results suggested that pupil dilation evoked by the emotional valence of auditory stimuli were mainly mediated by the sympathetic pathway, and similar pupil dilation was obtained between positive and negative valence stimuli.

To investigate whether the relationship between valence and evoked pupil dilation exhibited curvature while taking arousal level into account, as described previously for arousal analysis, we used a linear mixed model with valence rating (*V*), arousal rating (*A*), background luminance level (L) as well as the square of valence rating (V^2) as fixed predictors, and the dependent variable was mean pupil dilation:

$$\text{Pupil dilation} = \beta_0 + \beta_1 * V + \beta_2 * A + \beta_3 * L + \beta_4 * V * L + \beta_5 * V^2 + \beta_6 * V^2 * L \quad (2)$$

where β_i are the standard coefficients of the statistical model (intercept and slopes).

The results yielded β_1 = 0.0063, p = 0.81; β_2 = 0.022, p = 8.1e-23; β_3 = -0.17, p = 8.1e-06; β_4 = 0.012, p = 0.48; β_5 = -0.00063, p = 0.81; β_5 = -0.0009, p = 0.56, suggesting that only arousal and background luminance level significantly accounted for some fluctuations of mean pupil dilation on a trial-by-trial basis, and pupil dilation responses were not modulated by emotional valence. Together, these results suggested that valence level did not significantly affect pupil dilation evoked by emotional sounds.

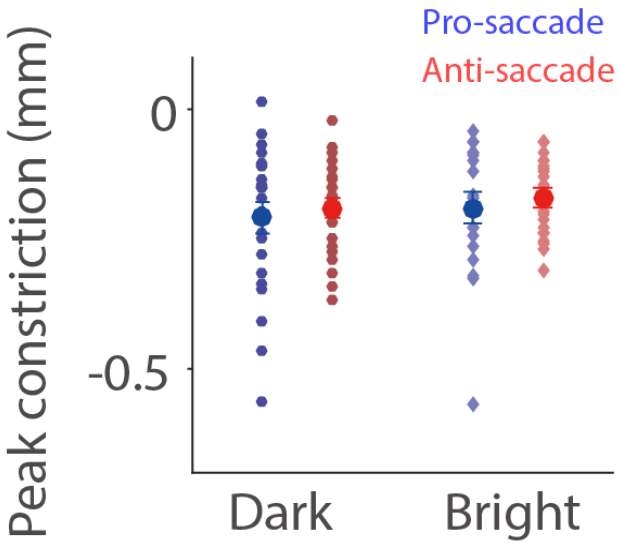

Supplementary Figure 3. Mean pupil sizes in peak constriction were -0.21 ± 0.030 and -0.19 ± 0.019 mm with the dark background, and -0.19 ± 0.025 and -0.17 ± 0.015 mm with the bright background in the pro- and anti-saccade conditions respectively. Although pupil size was larger in the bright than in the dark condition, the difference was not statistically significant ($F(1,22) = 2.675$, $p = 0.116$, $\eta^2 = 0.008$). Other effects were negligible (all *ps* > 0.17).

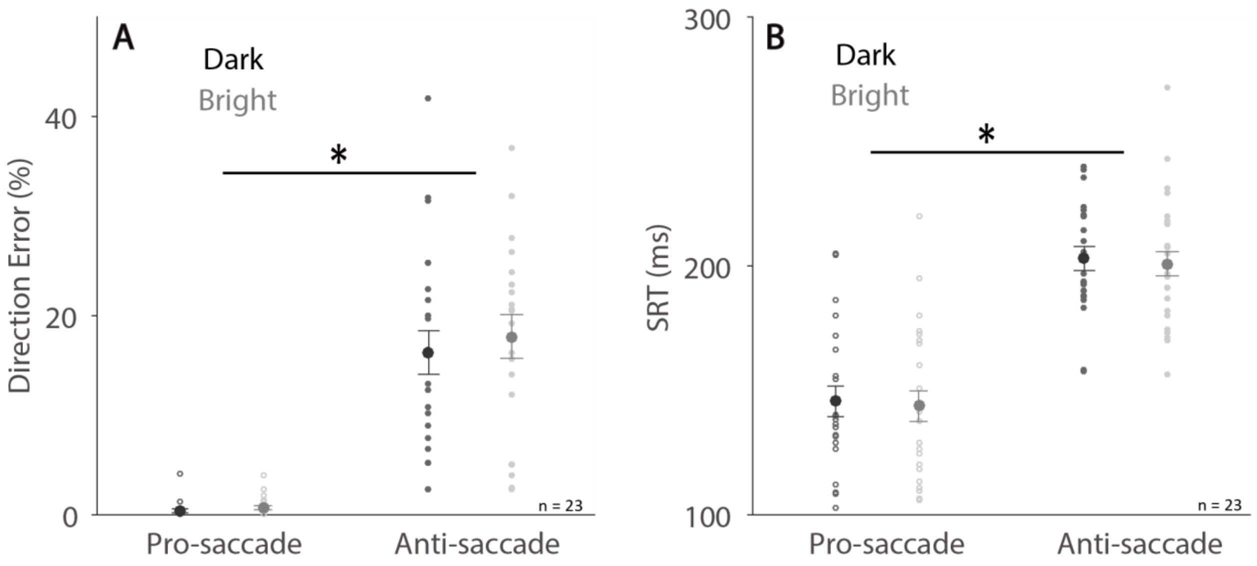

Supplementary Figure 4. Effects of background luminance on saccade behavior. Direction error (A) and saccade reaction time (B) in anti- and pro-saccade conditions (n=23). The large-circle and error-bars represent the mean values ± standard error across participants. The small circles represent mean value for each participant. * Indicates differences are statistically significant.

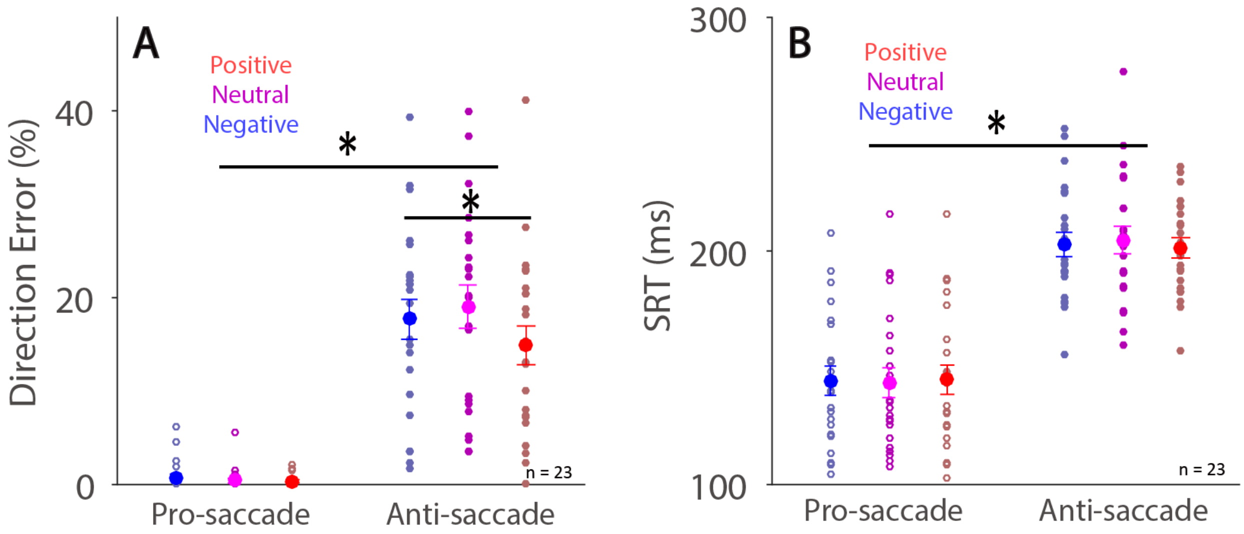

Supplementary Figure 5. Effects of emotional valence on saccade behavior. In addition to being modulated by saccade preparation (F(1,22) = 71.591, $p < 0.001$, $\eta^2 = 0.571$), direction error rates were significantly modulated by emotional valence (F(2,44) = 72.418, $p < 0.001$, $\eta^2 = 0.555$), with error rates of 0.770 ± 0.350, 0.422 ± 0.254, and 0.377 ± 0.155% in the pro-saccade trials, and 17.687 ± 2.094, 19.053 ± 2.274, and 14.905 ± 2.021% in anti-saccade trials in the negative, neutral, and positive valence conditions, respectively (sup Fig. 5A). The interaction was marginally significant (F(2,44) = 3.071, $p = 0.056$, $\eta^2 = 0.006$), the simple effects indicated a significant valence modulation in the anti-saccade condition (F(1,22) = 3.377, $p = 0.043$). SRT was only modulated by saccade preparation (sup Fig. 5B, F(1,22) = 190.182, $p < 0.001$, $\eta^2 = 0.550$), with mean SRTs in pro-saccade trials of 144 ± 6.097, 144 ± 6.264, and 145 ± 6.110 ms in the pro-saccade trials, and of 203 ± 5.108, 204 ± 5.744, and 201 ± 4.252 ms in anti-saccade trials in the negative, neutral, and positive valence conditions, respectively. No effects reached significance (all *ps* > 0.37). These results suggested that emotional valence modulated some saccade behavior.

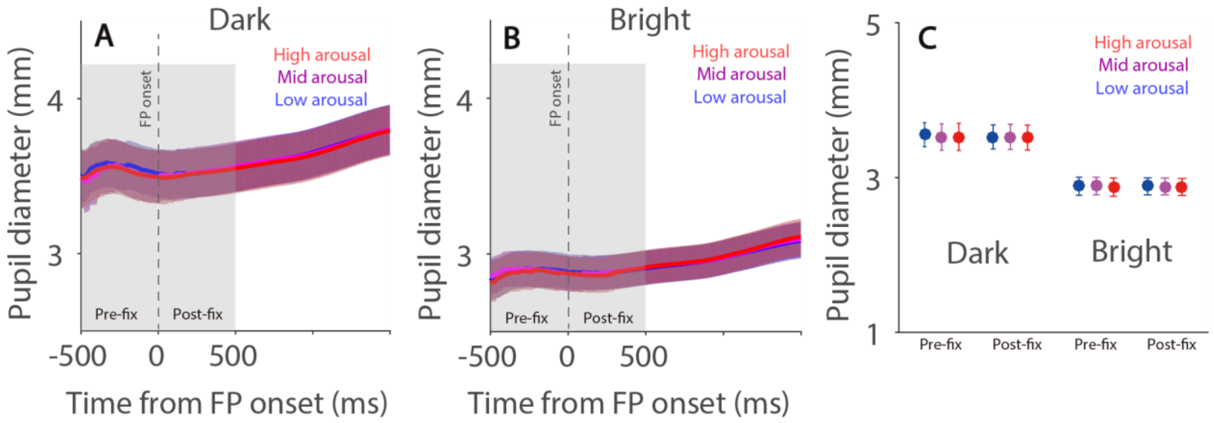

Supplementary Figure 6. Raw pupil diameter after central fixation point. Pupil changes in raw diameter following central fixation point (FP) presentation in different arousal conditions on dark (A) and bright (B) backgrounds (n=23). Mean pupil size at the pre-fix (-500 ms to FP onset) and post-fix (FP onset to 500 ms after it) epochs (C) of pupillary responses can be seen in different arousal conditions with dark and bright backgrounds (the circle and error-bars represent the mean values ± standard error across participants), showing similar pupil sizes in the pre- and post-fixation epochs (main effects of the FP epoch: Dark: $F(1,22) = 0.11$, $p = 0.743$, $\eta2 = 0.000$; Bright: $F(1,22) = 0.002$, $p = 0.963$, $\eta2 = 0.000$). Notably, prior to central fixation, pupil size significantly differed in the two background luminance conditions (main effects of background luminance in the pre-fix epoch: $F(1,22) = 36.326$, $p < 0.001$, $\eta2 = 0.195$). Together this suggests that prior to central fixation point presentation, the pupil has already reached a stable state.
